# Survey on Quality Of Life related factors in patients with Peptic Ulcer based on PRECEDE Model in Yazd, Iran

**Published:** 2011-11-24

**Authors:** MH Baghianimoghadam, S Mohamadi, M Baghianimoghadam, A Falahi, HS Roghani

**Affiliations:** Shahid Sadoughi University of medical sciences,YazdIran

**Keywords:** PRECEDE Model, Quality of Life, Peptic Ulcer

## Abstract

**Background:** Peptic ulcer is one of the most prevalent diseases. Its prevalence is estimated between 6-15% and, about 10% of the people, experience its symptom in a period of their life. Studies showed that quality of life and health status of peptic ulcer patients is affected by disease.

**Objectives:** The aim of this study was to determine the Quality of life Related Factors in patients with peptic ulcer based on Precede Model.

**Context:** A cross-sectional study with 120 subject patients, who were referred to Shahid Sadoghi Hospital of Yazd.

**Methods:** The patients were selected by simple Random sampling and data were collected by researchers making questionnaire. The validity and reliability of the questionnaire was approved. The data collected were transferred directly into SPSS. For data analysis, correlations, T–test, One way – ANOVA were used.

**Results and Conclusion:** Results showed that there was a significant difference between the Quality of Life and Self-Management (p=0.05) and Quality of Life with Health Status (p=0.01). Pearson correlation showed that there was a significant correlation between the Quality of Life and Reinforcing factors (P=0.01). Totally, it was found that the health status, Self–Management, Predisposing and Enabling factors predicted 0.64 of the quality of life variance, among which, the Health Status was the strongest predictor (β=0.575). What should be taken into account is the cultural situation of Iran and the fact that the PRECEDE model is a model for planning intervention. It can be used as a framework for planning intervention in order to promote the quality of life of ulcer peptic patients in Iran.

## Introduction

Peptic ulcer is defined as ulcers in stomach or duodenum and is usually recurrent [**1**]. Ulcer contains a mucosal lesion in stomach, pylorus, duodenum or esophagus that has a pitting outward [**2**]. Peptic Ulcer Disease (PUD) is one of the most important problems in medicine. The incidence of PUD in the United States is of 500000 cases annually. PUD recurrence is of about 4 million people annually [**1**]. Today we know that the main etiology of PUD originates from imbalance between invasive and defensive factors in the mucosa of stomach and duodenum. Clinical presentations includes dyspepsia (epigastric pain), GI discomforts like fullness, nausea, anorexia, weight loss, iron deficiency anemia and melena [**3**]. Disease can result in pain, bleeding and GI obstruction in some cases. Long life risk of PUD for each person is of about 5%–10%; then disease is important socially and economically. Based on new information the prevalence of PUD in men and women in United States is of 12% and 10% respectively. Additionally, the annual mortality reports, due to PUD complication, are of about 15000 cases [**4**]. In Iran, PUD is the 31st prevalent disease [**4**].

Several studies showed that the improvement in Quality Of Life (QOL) in patients with PUD plays an important role in the treatment of the disease [**5**]. It is notable that the Quality Of Life is a concept that must include all the somatic aspects, psychosocial functions, physical activities and other related factors of disease. A study indicated that QOL in PUD patients is lower than in normal population [**5**].

PRECEDE programming model presented by Gurin and Cutter (1999), highlighted a systematic process for designing, implementing and evaluating the health promotion programs for target groups. Picture 1 shows a schematic view of this process and includes all different factors that shape the health situation and also help the policy makers in having a concentrated set of causing factors for intervention goals. PRECEDE programming process was implemented for a wide spectrum of health promotion topics [**6**].

Quality Of Life is an effort that mankind do for a healthier and more creative life. This matter is related to everyone’s values. There are some influencing factors on the improvement of QOL, such as health status, predisposing factors (such as knowledge and attitude), reinforcing factors and behaviors of a person such as self management [**7**]. Health status is a concept that defines itself individually and everyone defines it according to its own criterion. This unique definition is influenced by different somatic and psychosocial factors. Self management is the key point in chronic diseases and improves skills for problem solving and self efficacy of patients and, also, patients can perform their knowledge in life [**7**]. One layer of the total three layers includes factors that shape the fourth step of PRECEDE model, represented by ecologic and educational factors. This step is superior to behavioral change because it makes reason and motivation for a behavior [**8**].

Reinforcing factors include given rewards and background that learners take from others after behavior and can reinforce or weaken that behavior after that [**6**]. Enabling factors are resources, skills or barriers that can help or prevent environmental or behavioral changes [**6**]. Considering the lack of studies about Quality Of Life and its related factors and the low level of Quality Of Life in developing countries like Iran, we implemented this study to determine the related factors of Quality Of Life among patients with PUD.

## Materials and methods

This cross–sectional study was done on 120 patients with PUD, who referred to the Gastroenterology Clinic of Shahid Sadoughi Hospital, in Yazd city (central part of Iran). Sampling was done randomly, based on the day of visit, between April–July 2009. Data were collected from patients with a confirmed diagnosis by a gastroenterologist. Data were collected by using a specially designed questionnaire. The questionnaire was funded on PRECECDE model structure including Quality Of Life, self management, health status, enabling factors, reinforcing and predisposing factors (including knowledge and attitude). Demographic variables were also collected. Questionnaires were completed in the form of an interview between researchers and patients.

The validity of the questionnaire was confirmed by a panel of experts. For confirmation of internal validity, the α–cronbach coefficient was determined as 95% for quality of life, 94% for health status, 75% for self management, 74% for reinforcing factors, 95% for enabling factors and 71% for attitude.

All the data were transferred to SPSS software and analyzed by chi–square and T–test Correlation coefficient. P–Values less than 0.05 were considered statistically significant.

The questionnaire consisted of 118 questions including 20 questions about the Quality Of Life (with valid score range of 0–60), 15 questions about self management (with valid score range of 15–75), 28 questions about the health status (with valid score range of 0–84), 8 questions about enabling factors (with valid score range of 8–32), 12 questions about reinforcing factor (with valid score range of 12–72), and predisposing factors that consisted of two parts, 12 questions (with valid score range of 0–32) about knowledge structures and 8 questions (with valid score range of 5–40) about attitude, and finally 15 questions about demographic variables. The structures of PRECEDE were evaluated by using the Likert scale`s 4, 5 and 6 levels.

## Results

From the total 120 patients included in study, 45 (37.7%) were men and 75 (62.2%) were women, between 14 to 80 years old, with a mean age of 39.18±16.33. 20.2% of the patients were single, 70.6% married and 9.2% divorced. Regarding the income, 60.5% of the patients had an income under 250$ and only 5% had an income more than 500$ monthly. About 62.2% of the patients had a family history of PUD in their first degree relatives. 20.8% of the patients had mild psychological problems, 19.2% anemia, 10.8% renal diseases and 11.7% hypertension.

Self management had a significant relationship with diseases (p=.03). **Table 1** shows mean, standard deviation, score range and mean percent of maximum score of PRECEDE model structure.

**Table 1:** Mean and SD and range of constructs of PRECEDE Model

Constructs of model	mean	SD	Minimum and maximum of scores	% mean of maximum
Predisposing factor	49.58	9.8	8–72	68.86
Enabling factors	22.66	3.6	8–32	70.81
Reinforcing factors	55.2	16.1	12–72	76.06
Self management	50.9	902	15–75	67.08
Quality of life	313.14	133.3	0–600	52.15
Health status	33.9	18.05	0–84	40.35

Data shows that the level of knowledge and attitude of patients was higher than mean scores. The score of knowledge was of 17.22%, the mean score was of 22% and the score of attitude was of 23.3%, out of 40. Based on the data, the scores of enabling factors, predisposing factors and reinforcing factors were higher than the mean scores but the score of health status was weak and lower than mean score (33.9 out of 84).

**Table 2** shows the relationship between the demographic variables and PRECEDE variables. After ANOVA test data indicated that there is statistically significant relationship between predisposing factors (p=.008), Quality Of Life (p=.001) and health status (p=.001).

**Table 2:** The relationship between the variables of study and demographic variables

Demographic variables	reinforcing factors	Predisposing factor	Quality of life	Enabling factors	Health status	Self management
Age	N.S	0.008	0.000	N.S	0.000	N,S
Sex	N.S	0.000	0.012	0.033	0.000	0.028
Job	N.S	N.S	0.000	N.S	0.000	0.05
Income	N.S	0.003	N.S	0.005	N.S	0.015
Education	N.S	0.000	N.S	0.000	N.S	N.S

There was a significant relationship between sex and quality of life, health status and predisposing factors.

Only the Quality of Life (p=.001) and health status had a significant relationship with the job, showing that the mean scores of this two variables were higher in retired patients than farmers. ANOVA test showed that there was a significant relationship between the health status and the level of graduation. Mean scores of patients with high school graduation and higher education were higher than in uneducated patients. Income had a significant relationship with QOL (p=.003) and health status (p=.005). Mean scores of health status and QOL were lower in patients with low income.

**Table 3** shows the coefficient correlation of health status, Quality of Life, self management, enabling factors, reinforcing factors and predisposing factors variables. There was relationship between QOL and self management and health status at the level of .05 and .01 respectively.

**Table 3:** The coefficient correlation of health status, Quality of Life, self management, enabling factors, reinforcing factors and predisposing factors

Constructs of model	Predisposing factors	Health status	Quality of life	Self management	Reinforcing factors	Enabling factors
Health status	1					
Quality of life	0.586	1				
Self management	0.131	0.191	1			
Reinforcing factors	0.179	0.526	0.147	1		
Enabling factors	0.224	0.307	0.292	0.043	1	
Predisposing factors	0.180	0.146	0.135	0.190	0.003	1

Based on regression test (**Table 4**) the degree of prediction in this study was done by structures of health status, self management, enabling factors, reinforcing factors and predisposing factors, which together sum up to 64%. Of these variables, the role of health status (β=.575) was more important; and, also, Pearson coefficient showed that there was a positive relationship between QOL and reinforcing, enabling and predisposing factors at the level of .01, .05 and .05 respectively.

**Table 4:** Regression analysis of quality of life and predisposing of constructs of PRECEDE Model

Constructs of model	B	SE	T	P	R2	Dependent variable
**Health status**	0.575	1.062	7.104	0.000		
**Self management**	0.016	1.15	0.196	0.845		
**Reinforcing factor**	0.043	2.89	0.541	0.590	0.64	Quality of Life
**Enabling factor**	0.186	0.649	2.344	0.021		
**Predisposing factor**	-0.004	1.05	-0.053	0.958		

## Conclusions

Based on our data, most of the patients were women (62.2%) aged 14–45 years old. There was a significant relationship between the Quality Of Life (QOL) and age, job, sex, income and the level of graduation, which confirms the study of Johnsen et al [**9**].

About the effect of demographic factors on QOL, we can conclude that planning educational programs needs more powerful strategies and also more specific interventions considering age, sex and generally demographic factors. In our study, the mean score of health status in men was better than in women, that this is not matched with other similar studies [**10**].

Results of our study show that self management has a significant relationship with the family history. Being a patient in a family can change the concept of others about a disease. They try to avoid risky behaviors such as eating spicy foods and pickles; they must try to improve the level of the patient’s knowledge through education. This will help the patient have an active and effective role in transmitting information to other family members.

Based on this study, the pattern of the patients’ answers to the QOL questions was near to the mean scores. We suggest education on disease related factors, techniques for patients’ education in hospital wards, improvement of effect and applicability of educational programs content, by using medical students, residents, nurses and by improving their skills and capabilities regarding their communication with the patients. The patients must have enough information about their disease.

Exercise has an important role in QOL and must be taken into consideration in health education programs.

There was a significant relationship between QOL and health status and self management. Other studies have shown that, with the improvement of health status, QOL will improve too [**11**]. Another study concluded that self management has the most important role in health providing, treatment and care of people [**12**], but our study shows that health status is more important in QOL. This study shows that health system policy makers must understand the importance of QOL related factors such as health status and self management; and, after that, they should plan detailed programs and interventions, specifically [**9**]. In this study, we reported that 20.8% of patients had concomitant psychological disorders, confirmed by Johnsen’s study, which noticed the impact of PUD on the patient’s mind [**9**]. It also confirms the data of another study, which reported that 60% of the patients believe that PUD is related to stress [**12**].

Considering the high prevalence of psychological disorders in PUD patients, compared with others, and, because of PUD presentations’ overlapping and some psychological disorders, the importance of providing psychological consultations and treatments and also psychologically based health education is obvious.

Knowledge rate of patients was higher than the mean score range and the attitude was according to mean score range. These data were in opposition to Bystrov study which concluded that the level of attitude in patients was not enough [**13**]; and is also against the result of another study, which believes that the knowledge of patients was improper [**14**].

We concluded that, with increasing knowledge, the attitude toward avoiding the risky factors will improve, then, with improving knowledge and also attitude, we can change the behavior toward treating the disease. Enabling factors were in mean scores range, this showing the effectiveness of social support, especially in family treatment and control of disease. In one study, Fukunishi showed that weak social support can increase the rate of PUD [**15**].

There was a significant relationship between reinforcing factors and QOL (p=.01) in our study. These data confirm the important role of friends and family in the treatment of the disease; then the interventional programs must be done for these persons. For example, the education of the patient’s attendants in hospital or office of physicians, about the effect of their support on the patient’s health, can be appropriate. This way we can take advantage of the health system employees. These programs do not imply extra costs for health managers, so these programs have the capability of integration in health care programs.

Based on this study we conclude that, considering the cultural situation of Iran, PRECEDE model could be the base of planning of intervention programs to improve the Quality Of Life (QOL) of patients with chronic diseases, especially with Peptic Ulcer Diseases (PUD).
